# Rethinking the potential role of dose painting in personalized ultra-fractionated stereotactic adaptive radiotherapy

**DOI:** 10.3389/fonc.2024.1357790

**Published:** 2024-03-20

**Authors:** Hao Peng, Jie Deng, Steve Jiang, Robert Timmerman

**Affiliations:** ^1^ Department of Radiation Oncology, University of Texas Southwestern Medical Center, Dallas, TX, United States; ^2^ Medical Artificial Intelligence and Automation Laboratory, University of Texas Southwestern Medical Center, Dallas, TX, United States

**Keywords:** radiotherapy, pulsar, dose painting, TCP, PET, MRI

## Abstract

Fractionated radiotherapy was established in the 1920s based upon two principles: (1) delivering daily treatments of equal quantity, unless the clinical situation requires adjustment, and (2) defining a specific treatment period to deliver a total dosage. Modern fractionated radiotherapy continues to adhere to these century-old principles, despite significant advancements in our understanding of radiobiology. At UT Southwestern, we are exploring a novel treatment approach called PULSAR (Personalized Ultra-Fractionated Stereotactic Adaptive Radiotherapy). This method involves administering tumoricidal doses in a pulse mode with extended intervals, typically spanning weeks or even a month. Extended intervals permit substantial recovery of normal tissues and afford the tumor and tumor microenvironment ample time to undergo significant changes, enabling more meaningful adaptation in response to the evolving characteristics of the tumor. The notion of dose painting in the realm of radiation therapy has long been a subject of contention. The debate primarily revolves around its clinical effectiveness and optimal methods of implementation. In this perspective, we discuss two facets concerning the potential integration of dose painting with PULSAR, along with several practical considerations. If successful, the combination of the two may not only provide another level of personal adaptation (“adaptive dose painting”), but also contribute to the establishment of a timely feedback loop throughout the treatment process. To substantiate our perspective, we conducted a fundamental modeling study focusing on PET-guided dose painting, incorporating tumor heterogeneity and tumor control probability (TCP).

## 1 Introduction

Traditional fractionated radiotherapy upholds two fundamental principles: daily fractionation and fixed treatment duration. These two principles leverage the disparities in radiobiological behaviors between malignant tumors and healthy tissues, striking a delicate equilibrium between tumor re-sensitization, regrowth, reoxygenation, resistance development, and the benefits of normal tissue repair (1–8). The two most employed models are the linear quadratic (LQ) model and the biological effective dose (BED) (9–11). However, a significant limitation in these models is the assumption of constant radiosensitivity throughout the treatment course. This neglects the consideration of inter- and intratumoral heterogeneity, and its dynamic evolution over time (12–17).

In addition to radiosensitivity, the efficacy of radiation therapy is influenced by its scheduling. Numerous inquiries have delved into the potential advantages of prolonging the treatment duration, employing both clinical studies and mathematical modeling. Examples include hypofractionated stereotactic radiotherapy, stereotactic radiosurgery, ramp-up scheduling, and intermittent therapy (18–29). Despite the challenges linked to testing a considerable number of permutations in terms of dose and timing combinations, multiple studies have indicated that non-uniform fractionation is at least as effective, if not superior, to standard treatment in terms of overall survival, tumor control, and time to progression (18–29). At UT Southwestern (UTSW), our focus has been on exploring Personalized, Ultra-fractionated Stereotactic Adaptive Radiotherapy (PULSAR). PULSAR leverages the precision provided by image-guided radiation therapy technologies, delivering ablative radiation doses in a pulse mode with extended intervals between pulses (up to weeks or months). Each patient’s response to treatment is distinct. PULSAR provides the flexibility to adapt to the evolving characteristics of tumor, encompassing changes in size, shape, and biomarker expression. To gain a clearer understanding of the unique characteristics of PULSAR, it is beneficial to contrast it with two counterparts: conventionally fractionated radiation therapy (CFRT) and standard stereotactic body radiation therapy (SBRT). In both CFRT and standard SBRT, it is not likely for the tumor to undergo substantial changes between two closely spaced radiation doses. Additionally, frequent dosing over brief intervals may not maximize potential synergies arising from concurrent immuno-oncology strategies. In a recent publication that delved into PULSAR in conjunction with anti-PD-L1 checkpoint inhibitor therapy using a preclinical model, the findings clearly indicated that the efficacy of immunotherapy was contingent upon the interval between two adjacent radiation pulses (30).

We believe there is a need to include non-uniform dose delivery in PULSAR, moving towards more personalized treatments. Firstly, advancements in imaging techniques, such as positron emission tomography (PET) with novel tracers and quantitative magnetic resonance imaging (MRI), provide more comprehensive anatomical, functional, and molecular information. On-board systems, exemplified by commercially available MR-Linac and PET-Linac, offer advanced image guidance and real-time adaptability. Secondly, the intervals between radiation pulses yield valuable images that, can form a feedback loop to evaluate the outcome of non-uniform dose delivery and make timely plan adaptation, with the aid of artificial intelligence (AI) and multiomics tools. Thirdly, PULSAR, as a special form of SBRT, minimizes the number of fractions with ablative dose per fraction (6-20 Gy). The increased dose, in contrast to the standard 2 Gy used in traditional daily fractionated radiotherapy, necessitates a more meticulous examination of the varied radiosensitivity effects. This is vital to address concerns associated with underdosing.

Dose painting is a technique that entails tailoring the radiation dose delivered to a specific area within a tumor (31–36). Instead of uniformly irradiating the entire tumor volume, dose painting allows for the adjustment of radiation doses to different regions inside the tumor based on factors such as the tumor’s biological/functional characteristics and response to treatment. Its ultimate objective is to enhance the therapeutic ratio by increasing the radiation dose to the most resistant or aggressive parts of the tumor, while sparing healthy surrounding tissues from unnecessary radiation exposure. By incorporating dose painting into treatment planning, radiation oncologists can devise highly individualized and targeted radiation plans for each patient. Note that there is a subtle distinction between dose painting and dose escalation. Dose painting primarily entails customizing the radiation dose distribution within the tumor based on its heterogeneous biological characteristics, while dose escalation focuses on uniformly increasing the radiation dose across the entire tumor.

## 2 Intratumoral heterogeneity and TCP modeling

PET, as a molecular imaging modality, offers various biomarkers for this purpose. For instance, it leverages fluorodeoxyglucose (FDG) to identify glucose metabolism and tumor malignancy, as well as fluoromisonidazole (FMISO) to detect hypoxic conditions (37, 38). It is important to note that FDG specific uptake value (SUV) alone, which measures glucose metabolism, does not fully represent tumor size and heterogeneity. Furthermore, its quantitation is influenced by factors such as inflammation and necrosis. On the other hand, FMISO PET is used to detect areas of low oxygen concentration, which are associated with increased resistance to radiation therapy. FMISO images are typically analyzed using metrics like *SUV_max_*, tumor-to-muscle ratio (*TMR*), or tumor-blood ratio (*TBR*). In the case of hypoxic tumors, it often requires a 2.5- to 3-fold higher dose to achieve an equivalent cytotoxic effect compared to non-hypoxic regions (39).

Over the past few years, the research community has encountered a blend of perplexing yet promising discoveries in relation to FDG/FMISO-based radiotherapy (40–46). One major question is the degree of spatial correlation between FDG and FMISO signals. FDG uptake hinges on the upregulation of glucose transporters and glycolytic enzymes, both of which are driven by hypoxia-inducible factor-1 transcription. As a result, this complicates the correlation between FDG-PET and FMISO-PET signals. For instance, Torwarth et al. found no correlation in 12 primary HNC tumors (41). Gagel et al. suggested that using FDG to discriminate hypoxia was not feasible, yet a strong correlation was evident between FMISO and the hypoxic condition within the range of 2.5 to 10 mmHg, as determined through polarographic needle electrode measurements (42). Nehmeh et al. demonstrated that the voxel-wise correlation between FDG and FMISO is moderate within primary tumors, but no correlation exists in lymph nodes (45). Another relevant question is whether FDG/FMISO has any prognostic value in radiotherapy. Watanabe et al. discovered that in non-small cell lung cancer (NSCLC) patients, those with both high FDG and high FMISO levels exhibited the poorest prognosis (46). FDG uptake emerged as a good predictor of short progression-free survival (PFS) only in patients of small tumors (<2 cm). In contrast, FMISO uptake consistently acted as a better predictor of short PFS in all patients, irrespective of tumor size. In the case of head and neck squamous cell carcinoma (HNSCC) patients undergoing chemo-radiation, Lee et al. reported no dependency of treatment outcomes on the hypoxic status of tumors (43), whereas Nicolay et al. presented opposite findings (44).

**Figure 1:**
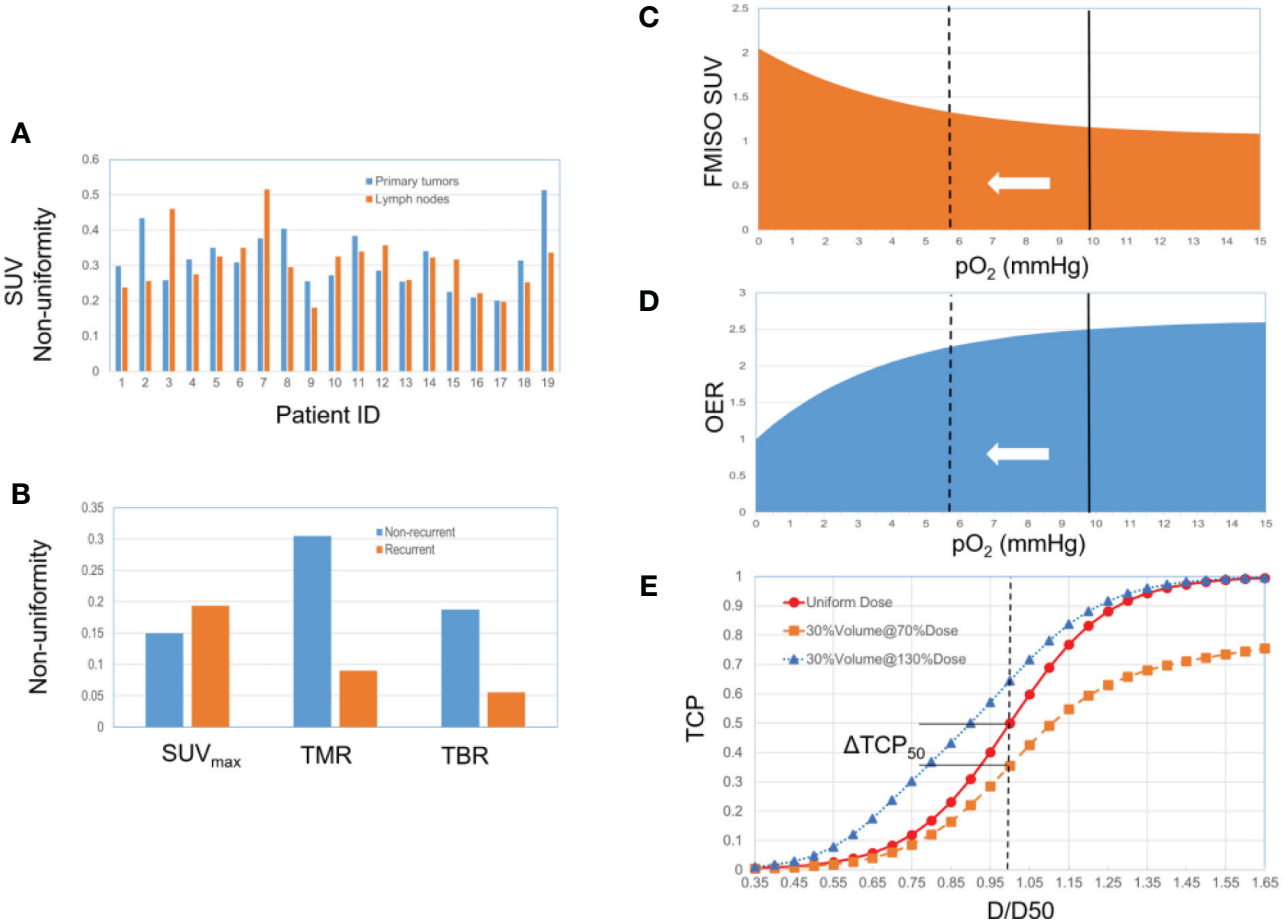
**(A)** 19 HNSCC patients, primary tumors and lymph nodes (from ref 45). **(B)** 22 NSCLC patients, non-recurrent group (13 patients) and recurrent group (9 patients) (from ref 46). **(C)** Relationship between FMISO SUV and pO_2_. The solid line represents the selected baseline reference (pO_2_ = 10 mmHg). The dotted line represents the hypoxic state, corresponding to a higher SUV and a lower OER. **(D)** Relationship between OER and pO_2._ **(E)** The TCP dose-response curve (a sigmoid function) changes along with the hypoxic condition and associated radio-sensitivity.

Tumor heterogeneity arises from two sources: the non-uniformity of SUV and the percentage of hypoxic volume relative to total tumor volume. To gauge the potential benefits of dose painting, we developed a simple TCP model as described below. The distribution of non-uniform FMISO SUV is illustrated in **Figures 1A, B**, drawing from two recent publications that center on FDG/FMISO-based SBRT. One study explored the tumor non-uniformity and the quantitative relationship between FDG and FMISO in a patient cohort, encompassing 20 primary tumors and 19 metastatic lymph nodes (45). The FMISO SUV non-uniformity (per patient) ranges from 18% to 52%. In another study of NSCLC patients treated with SBRT, the FMISO PET non-uniformity (recurrent/non-recurrent groups) reaches up to *SUV_max_* (14%/19%), TMR (30%/9%), and TBR (18%/5%) (46). Besides non-uniform *SUV*, estimating the volume percentage of the hypoxic regions necessitates a region-of-interest approach, with limited published data available. In a study with a cohort of 323 patients across 17 different studies, the ratio between the hypoxic volume and the total tumor volume was estimated to fall within the range of 34% and 42% (40). Additionally, it was observed that with each doubling of the tumor volume, the fraction of hypoxic volume increased by four percentage points (41). As a result, we evaluated the following two extremes representing the hypoxic volume fraction, 25% and 50%.

Our simplified model consists of three steps as depicted in **Figures 1C-E**. First, the quantitative relationship between FMISO SUV and pO_2_ was established based on an empirically fitted model (organ site-specific) (47) (Equation 1 and **Figure 1C**). Second, the oxygen enhancement ratio (OER) as a function of pO_2_ was modeled (48–50) (Equation 2 and **Figure 1D**). The baseline reference of the partial pressure of oxygen associated with *SUV* was arbitrarily selected to be 10 mmHg. OER reflects the difference in the required dose corresponding to a given survival fraction, between two oxygenation conditions. A hypoxia-equivalent dose can thus be derived, scaling down by the OER relative to the baseline. Third, the TCP dose-response was modelled considering the heterogeneity of hypoxic condition, based on a logistic function (Equation 3) (51–53). The TCP depends on two parameters, D50 (the dose corresponding to a TCP of 50%) and γ50 (the percent change in TCP per percent change of dose at the point at D50). A constant SUV was assumed inside the hypoxic volume with the uptake of *SUV_max_*, with the non-uniformity defined as (*SUV_max_-SUV_avg_*)/*SUV_avg_*. We subsequently combined the dose responses of both hypoxic and non-hypoxic volumes to create the overall TCP curve, using a population-averaging approach (54, 55). D50 was set to be 80 Gy. Three γ50 values were evaluated (3, 2 and 1), representing steep, moderate, and shallow dose-response relations, respectively. Finally, the absolute change in TCP was quantitatively analyzed using two figures-of-merit (FoMs): ΔTCP_50_ and ΔTCP_80_.


(1) $$FMISO\;SUV=1.05+6.7{\;}^{\left(-0.117pO_{2}\right)}$$

(2) $$OER\left(pO_{2}\right)=1+1.63\left(1-e^{-0.26\;pO_{2}}\right)$$

(3) $$TCP\left(D\right)=\frac{1}{1+\text{exp}\left(-\left(D-D50\right)/k\right)}\;\;\;\;\;\;k=\frac{D50}{4\gamma_{50}}$$

One important characteristic of the TCP dose-response is illustrated in **Figure 1E**. The heterogeneity, either hypoxic condition or other biological characteristics, results in varying radiosensitivity. Under a uniform dose delivery, this implies that some spots are under-dosed (e.g., 50% of the dose to 30% of the volume), and the TCP curve is lowered consequently. The same argument applies to a situation in which the clonogen’s radiosensitivity is uniform, but dose delivery is non-uniform. A distinctive characteristic of the sigmoid curve is that the under-dose region holds greater significance than the over-dose region, while over-dosing the non-hypoxic sub-volume has a minor impact. Even when the hypoxic sub-volume constitutes a small fraction of the total tumor volume, it exerts a dominant influence on the TCP profile. If the hypoxic sub-volume can be identified through techniques like PET or MRI, dose painting strategies can thus be implemented accordingly to restore the TCP curve.

**Figure 2:**
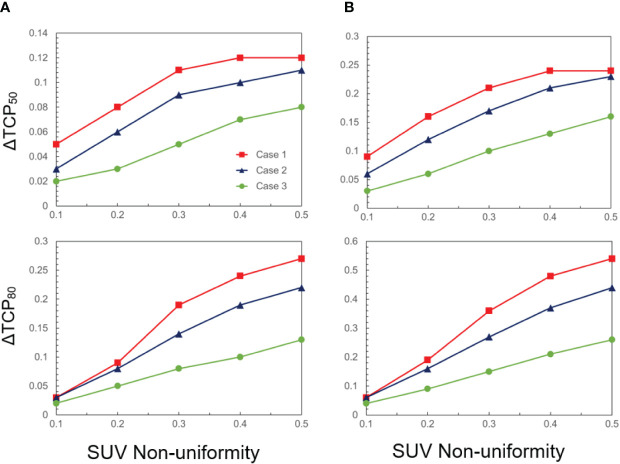
ΔTCP_50_ and ΔTCP_80_ as a function of SUV non-uniformity for three cases (case 1: γ50 = 3, case 2: γ50 = 2, case 3: γ50 = 1), and two hypoxic volume fractions: **(A)** 25% and **(B)** 50%.

The quantitative analyses of the change in both ΔTCP_50_ and ΔTCP_80_ are summarized in **Figure 2**, as a function of FMISO SUV non-uniformity. All cases exhibit a linear relationship with dose non-uniformity. For a larger hypoxic sub-volume and a steeper dose-response curve, the dependency is stronger. For a relative volume of 50% and γ50 = 3, ΔTCP_50_ and ΔTCP_80_ amount up to 24% and 54%, respectively. On the other extreme, for a relative volume of 25% and γ50 = 1, both ΔTCP_50_ and ΔTCP_80_ are about 2%. Note that while TCP is often discussed in the context of populations to evaluate treatment strategies, it can also be applied individually to inform treatment decisions for specific patients.

## 3 Discussion

Below we discuss the potential needs and benefits of conjoining dose painting with PULSAR. The combination of the two may not only provide another level of personal adaptation, but also contribute to the establishment of a timely feedback loop throughout the whole treatment. A mechanistic model that incorporates parameters such as SUV, OER, and TCP is presented. Despite the simplicity of the model and crude borderlines, it may serve as a guideline for designing dose painting studies and interpreting experimental results. The parameters in Equations 1-3, as well as the baseline reference associated with SUV and OER, can be flexibly adjusted for different imaging protocols or diseases sites. More advanced computational modelling of the tumor microenvironment can enable the design of virtual clinical trials. Nevertheless, as stated in the Introduction section, although PULSAR is a very recent concept, dose painting has been in place for many years with limited clinical adoption. In our view, controversies surrounding PULSAR and dose painting will persist, giving rise to numerous research questions to be answered. Two unique aspects along with several practical considerations, are elaborated below.

The first aspect pertains to the role of imaging in treatment planning and the spatial extent to which dose modulation can be achieved. This aspect is also closely connected to the distinction between dose painting and dose escalation. First of all, we need more evidence to determine whether dose painting (combined with more specific biomarkers) will surpass nonselective dose escalation and achieve improved treatment outcome (37). In addition, challenges exist regarding the utilization of information on tumor heterogeneity derived from PET and MRI images. Although the FMISO uptake represents the hypoxic heterogeneity inside tumors and identifies a biological target volume, it may not be suitable for contouring purposes. The target volumes such as gross tumor volume (GTV) and clinical target volume (CTV), are routinely based on FDG PET. Note that the spatial information of heterogeneity is not reflected in **Figures 1A, B**, and the TCP change derived in our model assumes that the hypoxic-related heterogeneity comes from FMISO alone. However, such heterogeneity may be attenuated by the FDG-associated heterogeneity. For instance, a dose-painting strategy relying on dose escalation to the hypoxic region only, would not be advantageous if the hypoxic region extensively overlaps with the hyper-metabolic volume based on FDG-avid voxels (45). That said, it is the additional contrast between FMISO and FDG that determines the potential benefits of dose painting. In comparison with FDG-PET, the slower pharmokinetic of FMISO (~3-4 hours) and lower SUV (~1-3) may introduce additional uncertainty in quantification which should also be considered in dose painting (45, 46). Other PET tracers for imaging hypoxia can also be examined for PET-guided dose painting, such as HX4, ATSM and FAZA (56–59). Lastly, we anticipate that immuno-PET has great potential to open unique research avenues and foster the synergy between PULSAR and immunotherapy (60).

Quantitative MRI represents another valuable imaging tool in dose painting. Within tumors, subvolumes exhibiting distinct functional characteristics, such as physiology, perfusion, and oxygenation, can be recognized as diverse tumor habitats (61–64). For example, in glioblastoma, hypercellular tumor components identified through high b-value diffusion-weighted imaging (DWI) offer additional insights compared to those from dynamic contrast-enhanced (DCE) (63, 64). The hypercellular tumor volume determined by diffusion MRI well correlates with progression-free survival (61). Therefore, the thresholds of quantitative MRI imaging features can be determined empirically or established through histopathology and clinical outcome. Along with PET, they hold significant promise to provide radiation oncologists with more insights for crafting dose painting strategies.

From a physics and engineering standpoint, the spatial modulation capacity of beam delivery and factors such as finite image spatial resolution (e.g., 4-6 mm) may impose constraints on the implementation of dose painting. This is reminiscent of the situation in spatially fractionated or GRID radiation therapy, a specialized technique that administers high-dose radiation in a grid-like or checkerboard pattern to the target area, with a modulation capacity of approximately 1 cm (65). In conventional radiotherapy, the multi-leaf collimator offers spatial modulation with a resolution of ~5 mm. Analogously considering the concept of point spread function in image reconstruction, it prompts an intriguing question of whether the attainable benefits of dose painting might be attenuated, compared to the results shown in **Figure 2**. Additional challenges in margin setting and motion management contribute to the concerns surrounding this issue.

The second aspect pertains to the extended interval between fractions, a unique feature of PULSAR. Biology takes time. Longer intervals allow more normal tissue recovery after injury, and at the same time, provide time for tumor to undergo dramatic changes including radioresistance. PULSAR can also operate in association with immunotherapy. Compared to daily or every other day fractions, PULSAR is more likely to harness the synergy between the two, since the adaptive immune response takes time to develop and reach its full effectiveness (30, 66–70). Recently, several modeling studies have been conducted to provide mechanistic interpretation of the interaction of radiation with checkpoint blockers through ordinary differential equations, examining the dependence of the PULSAR effect as a function of dose and timing (71–73).

We recognize that the existing evidence does not entirely endorse the effectiveness of dose painting, and skepticisms persist in a number of aspects including the reproducibility of radiotracers, the need of intra-fractional PET, and the consistency of radiotracers in characterizing chronic hypoxia during radiotherapy. Notwithstanding these concerns, we envision that dose painting has potential to be integrated with PULSAR to address intratumoral heterogeneity and improve overall treatment response, targeting locoregional-resistant regions more effectively (74). Three scenarios are elaborated below.

Firstly, PULSAR minimizes the number of fractions with ablative dose per fraction (6-20 Gy). Compared to 2 Gy in traditional daily fractionated radiotherapy, the anticipated impact of dose modulation will be magnified, due to the shift toward the high-dose end in the survival curve as described by the LQ model. In other words, a large dynamic range is available for implementing dose painting and mitigating possible underdosing.

Secondly, the extended interval in PULSAR allows for more complete reoxygenation. The process of reoxygenation itself is rapid, but its cumulative impact on the hypoxic state post-irradiation is complicated and may last several days. One single fraction SBRT study reported that the proportion of surviving hypoxic cells decreased over several days postirradiation (75). One plausible explanation is that the oxygen supply through small fractions of blood vessels that survived high-dose exposure, combined with a reduction of oxygen consumption due to massive cell death, causes the reoxygenation of hypoxic cells (75). In PULSAR, the extended inter-fraction time helps ensure complete reoxygenation while avoiding repopulation. Dose painting can thus be implemented based on the latest distribution of hypoxic sub-volumes, improving the efficacy of radiation treatment.

Thirdly, multiple PET or MRI images are accessible at various stages in PULSAR (pre-treatment, mid-treatment, and post-treatment), which provide valuable insights into patient response to treatment. These data can be analyzed using multiomics (radiomics, dosiomics) and AI tools. For example, delta radiomics entails the extraction of radiomic features from the same region of interest within the same patient but at different time points (76–79). In contrast to standard radiomics, which captures a static snapshot, delta radiomics focus on the phenotypic changes in tumors that occur after the treatment. It is thus reasonable to expect that delta radiomics will provide enhanced predictive capabilities compared to conventional radiomic features, making them especially suitable for PULSAR. Besides radiomics, other deep learning features such as U-Net or transformer-based models can also be used to extract specific architecture or details within the images. In the end, we can adopt a more personalized approach based on AI prediction of each patient’s distinct response. Moreover, dose painting in PULSAR may go beyond pre-treatment images and incorporate post-treatment images for updating dose prescription, akin to background subtraction. The adaptive dose painting, in conjunction with a concurrent feedback loop through multiple imaging steps, allowing us to assess both the need and efficacy of dose painting in a timely manner. On a side note, the combination of PULSAR with dose painting can play a unique role in both photon and particle therapy. One promising direction can be the use of carbon or, even better, oxygen ions to overcome hypoxia independently of dose painting (80, 81).

To conclude, we foresee that the advancements in imaging technology, the emphasis on adaptive radiotherapy, the evolution of AI tools, and combined radio-immunotherapy are bolstering the demand for dose painting. The integration of dose painting into PULSAR, while a novel concept, is expected to hold substantial promise for progressing radiation therapy, initiating a shift in the approach to personalized cancer treatment.

## Data availability statement

The raw data supporting the conclusions of this article will be made available by the authors, without undue reservation.

## Author contributions

HP: Investigation, Resources, Writing – original draft, Writing – review & editing. JD: Writing – review & editing. SJ: Writing – review & editing. RT: Writing – review & editing.
